# Trend Patterns of Vegetative Coverage and Their Underlying Causes in the Deserts of Northwest China over 1982 – 2008

**DOI:** 10.1371/journal.pone.0126044

**Published:** 2015-05-11

**Authors:** Tianyi Zhang, Hesong Wang

**Affiliations:** 1 State Key Laboratory of Atmospheric Boundary Layer Physics and Atmospheric Chemistry, Institute of Atmospheric Physics, Chinese Academy of Sciences, Beijing, China; 2 Key Laboratory for Silviculture and Conservation of Ministry of Education, College of Forestry, Beijing Forestry University, Beijing, China; University of Vigo, SPAIN

## Abstract

We identified the spatiotemporal patterns of the Normalized Difference Vegetation Index (NDVI) for the years 1982–2008 in the desert areas of Northwest China and quantified the impacts of climate and non-climate factors on NDVI changes. The results indicate that although the mean NDVI has improved in 24.7% of the study region; 16.3% among the region has been stagnating in recent years and only 8.4% had a significantly increasing trend. Additionally, 45.3% of the region has maintained a stable trend over the study period and 30.0% has declined. A multiple regression model suggests that a wetter climate (quantified by the Palmer Drought Severity Index, PDSI) is associated with higher NDVI in most areas (18.1% of significance) but these historical changes in PDSI only caused an average improvement of approximately 0.4% over the study region. Contrasting the regression results under different trend patterns, no significant differences in PDSI impacts were detected among the four trend patterns. Therefore, we conclude that climate is not the primary driver for vegetative coverage in Northwest China. Future studies will be required to identify the impacts of specific non-climatic factors on vegetative coverage based on high-resolution data, which will be beneficial in creating an effective strategy to combat the recent desertification trend in China.

## Introduction

Desert ecosystems are particularly vulnerable to water availability and human intervention. In China, approximately 173 million ha of desert (58% of this land) is located in the arid climate of Northwest China [1]. Any potential signs of desertification are of great concern in China in the context of climate change [2] and the overexploitation of local natural resources [3].

Climate change has significant influences on the vegetation growth [4, 5]. Some studies, based on water and energy balance models, have suggested that water resources in northwest China have been overexploited, reducing the stability of dryland ecosystems [6] and resulting in desertification [7]. However, there is abundant evidence that China is under a process of greening in previously degraded areas [8–10]. For example, Normalised Difference Vegetation Index (NDVI) data showed a linear increasing trend during the period of 1982–1999, indicating progressively improving vegetative coverage and declining desertification in Northwest China [5]. A more recent study found that NDVI has tended to decline since 1998, despite the long-term increasing trend [11]. This finding is a potential signal that may reflect increasing negative pressures on the dryland ecosystems in recent years.

By geographically contrasting the spatial distribution of NDVI trends with climate trends [11] or by applying a simple correlation analysis of these trends [5, 12], many studies have attributed the overall improvement of the NDVI to the progressively wetter climate in arid areas since the 1980s. However, it is also important to bear in mind that climate is not the only driver. Other non-climatic factors, particularly human influences like applying conservation policies, may also lead to improved vegetative coverage [13, 14]. Because there is no consensus on the primary driver for the changes in vegetative coverage, it is difficult to develop an effective policy to achieve the trade-off between economic gains and environmental protection in the desert areas of China.

The objective of this study was to quantify the greening of the landscape due to vegetative recovery and assess the underlying factors driving revegetation in the desert areas of Northwest China between 1982 and 2008. In contrast to earlier assessments, our conclusions are reached by combining two analyses: trend patterns and multiple regression analysis. The trend pattern analysis identifies the NDVI trends patterns. The multiple regression analysis assesses the impact of the individual climate and non-climatic factors on NDVI.

## Materials and Methods

### Study region and data

**Fig 1:**
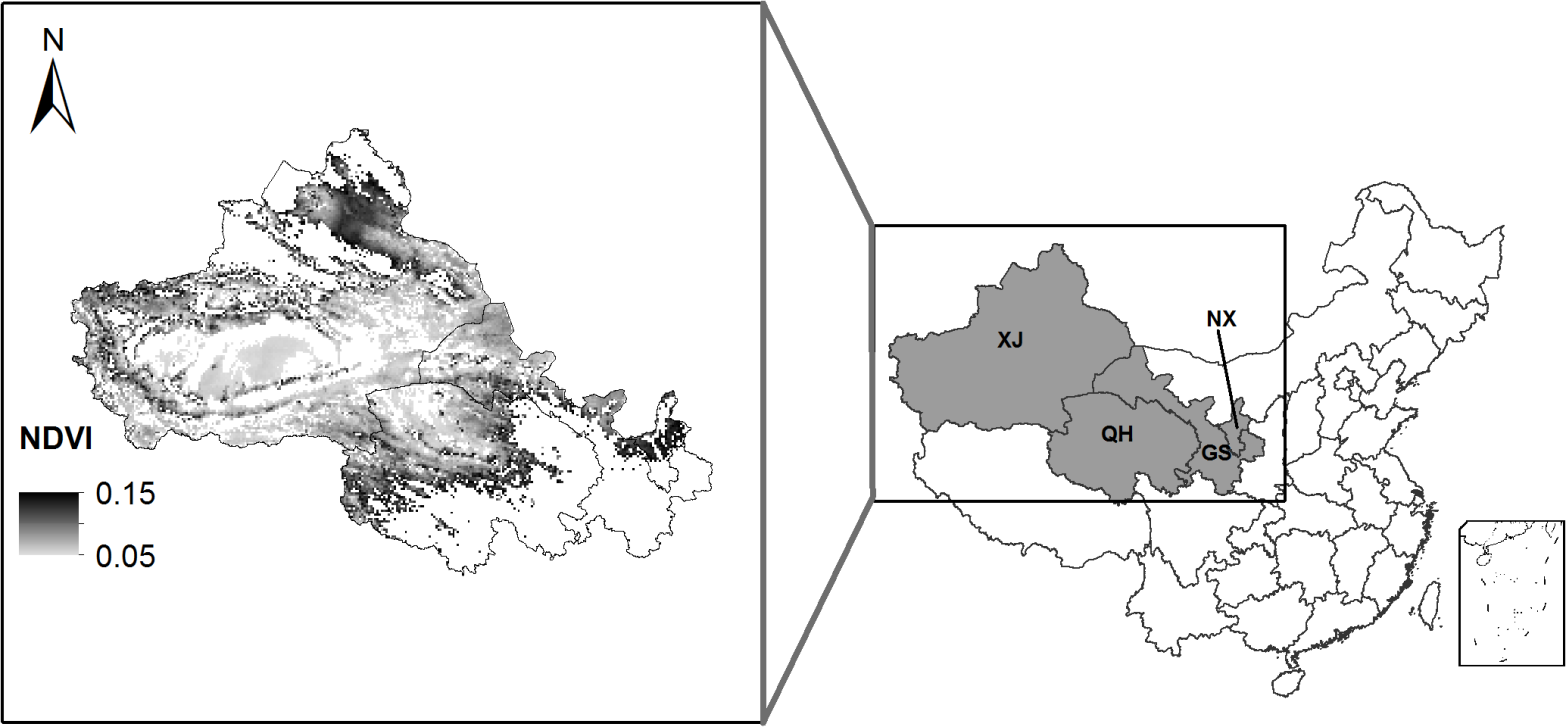
Study region. Four provinces were included: Xinjiang (XJ), Qinghai (QH), Gansu (GS) and Ningxia (NX). We concentrated on areas with long-term average NDVI values between 0.05 and 0.15. The base map is only for illustrative purposes.

We focused on four provinces (Xinjiang, Qinghai, Gansu and Ningxia) located in northwest China (Fig 1) that have a typical arid climate with approximately 290 mm annual precipitation and approximately 1200 mm annual evapotranspiration. Approximately 100.3 million ha of desert lands are located in these four provinces [1].

NDVI is defined as the ratio of the difference between near-infrared reflectance and red visible reflectance to their sum, and has been an indicator often used to quantify vegetative coverage on regional [5] and global scales [12]. A NOAA Global Inventory Modelling and Mapping Studies (GIMMS) NDVI dataset for the period from January 1982 to December 2008 was obtained from original NOAA AVHRR data as bi-weekly maximum value composites aggregated to an 8 km resolution [15]. A series of correction procedures for removing the noise due to sensor degradation, clouds and volcanic eruptions has been performed on the dataset, which allows for the assessment of NDVI trends on a long-term scale [16]. Annual average NDVI was calculated for each grid and used as an indicator of vegetative coverage in our study. We concentrated on those grids that had a long-term (1982–2008) average NDVI value between 0.05 and 0.15 (Fig 1), which were considered desert areas [11]. Thus, the NDVI metric primarily reflected changes of vegetative coverage in the existing desert areas.

Climate data were obtained from the China Meteorological Administration (cdc.cma.gov.cn), including the daily minimum and maximum temperatures, sunshine hours, wind speed and relative humidity at 756 land-based methodological stations distributed throughout China. For each day, an interpolation algorithm reported by Thornton et al. [17] was used to estimate the daily climate data (Fig 1). Soil information in our study was extracted from the grid soil [18]. Based on the database, we calculated the total available water, defined by the amount of water available between field capacity and the wilting point water contents, over the depth of 100 cm in the soil profile.

Since water availability is the primary climate factor for vegetative coverage in the drylands of the world [12], we calculated a widely-used drought indicator, the Palmer Drought Severity Index (PDSI) [19], to represent the water availability condition in each year. Earlier studies used precipitation as a measure of the aridity of the climate to inform desertification trends [2, 20], but this approach may overlook the influence of other important climate determinants of drought [21]. The PDSI is a multi-scalar drought index which incorporates the moisture supply (precipitation, Prcp) and demand (potential evapotranspiration, PE) in a two-layer-soil-bucket hydrological model [22]. Higher PDSI indicates wetter climate and lower PDSI indicates dryer climate conditions. Our PDSI calculation was based on an algorithm adapted from the National Climatic Data Centre [23], and we replaced the original Thornthwaite model [24] with the Penman-Monteith model [25] to calculate PE because the Penman-Monteith model provides more reliable PE estimates [26]. To match with the annual NDVI value above, we calculated the annual average value for PDSI.

### Statistical analyses

**Fig 2:**
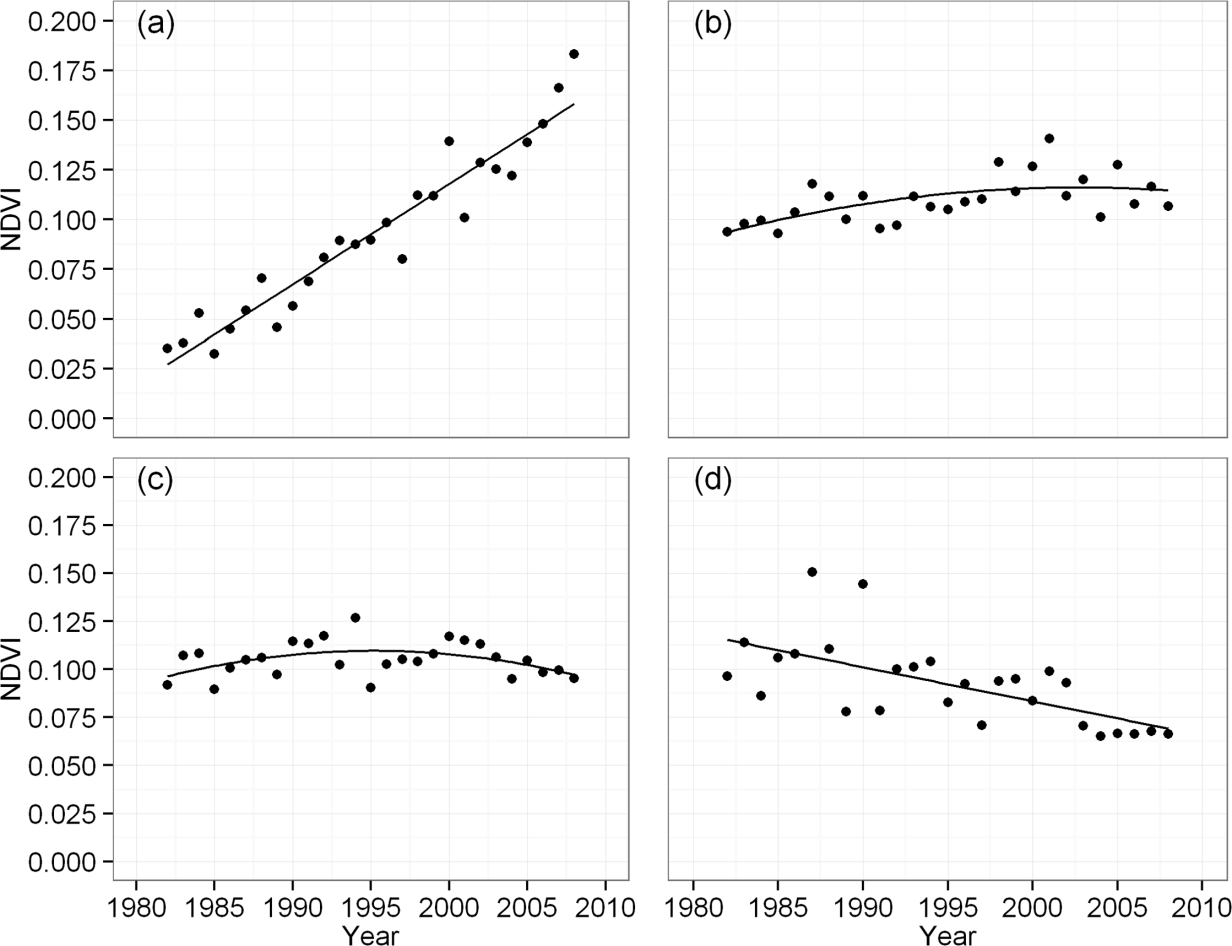
Examples of trend patterns of the NDVI. Increasing (a), stagnation (b), stable (c), and decreasing (d).

Firstly, we categorized the time series of each grid to one of four trend patterns, i.e., increasing, stagnation, stable and decreasing. The increasing pattern denotes a significant increasing trend of NDVI over study period (Fig 2A), the stagnation pattern denotes NDVI has been stagnating recently despite an overall improvement (Fig 2B), the stable pattern indicates NDVI has been maintaining a stable level over study period (Fig 2C), and decreasing pattern represent NDVI shows a significant decreasing trend over time (Fig 2D). To identify them, four regression models (intercept-only model, linear model, quadratic model and cubic model) were first fitted to the time series of each grid. The model with the best fit was selected based on the Akaike Information Criterion [27]. The goodness of fit was determined using an F-test, and the fitted parameters indicated the categories of NDVI trend patterns. The detailed processes of the identification of NDVI trend patterns are presented in S1 File and demonstrated by S1 Fig.

Secondly, to identify the climatic impacts on NDVI, a multiple regression model was applied to NDVI and PDSI data for each grid (Eq 1):

$$\text{log}({\text{NDVI}}_{t})=\beta_{0}+\beta_{1}{\text{PDSI}}_{t}+\beta_{2}{\text{Year}}_{t}+\varepsilon \;,$$ (1)

where log(NDVI_*t*_) is the natural logarithm of the annual average NDVI value in the year *t*. The reason for transforming to log(NDVI_*t*_) is to keep the NDVI variance comparable in relative rather than absolute terms. The regression coefficient can be interpreted as a given change in a certain independent variable that results in the same per cent change in the NDVI. In this equation, *β*_0_ and *ε* are the intercept and the error terms, respectively; PDSI_*t*_ is the annual average PDSI value in each Year *t* and *β*_1_ is the regression coefficient of the PDSI value, denoting the impact of climatic moisture conditions; Year_*t*_ is the observed year and *β*_2_ is the regression coefficient of Year. The Year_*t*_ term is a proxy indicator to capture the non-climatic impacts. For example, both of negative (e.g. overexploiting water) or positive (e.g. launching anti-desertification programs) influence could be associated with time trend, leading to lower or higher vegetative coverage and omitting it could result in potential bias in PDSI coefficients.

The uncertainty of the PDSI and Year impacts due to the limited sample size were evaluated by the bootstrap re-sampling approach. In each time series, years were randomly chosen with replacement. Then, we estimated the regression coefficients using the above regression model, and this process was repeated 1000 times to obtain the confidence interval. The confidence interval is calculated as the 2.5th and 97.5th percentiles of the coefficients obtained from the re-sampling procedure. The confidence interval not spanning zero indicates a significant effect.

To estimate the changes in NDVI induced by the historical trends of the PDSI and the Year during the study period, we input the historical data for PDSI and Year into the multiple regression model. After that, we compared the differences of the individual impacts on each trend patterns. The difference among patterns was assessed by Tukey’s honestly significant difference (HSD) test with P<0.05 considered to be statistically significant. If PDSI is the major driver for NDVI trend, we may expect a statistically significant difference for PDSI impacts among the four trend patterns.

## Results

### NDVI trend patterns

**Fig 3:**
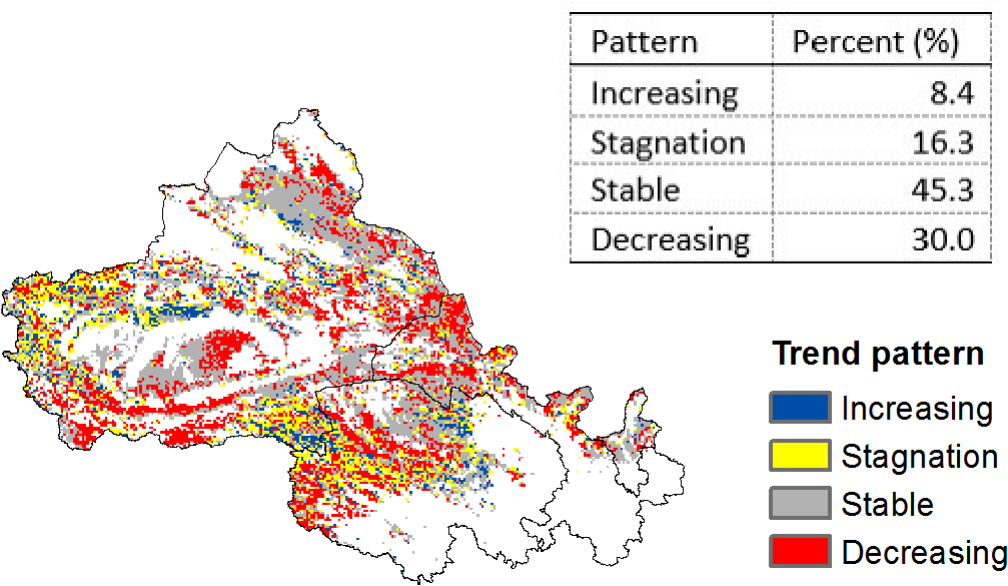
Trend patterns of the NDVI in the study region. The base map is only for illustrative purposes.

Based on the aforementioned four trend patterns, the results show a stable time trend of NDVI in approximately 45.3% of the area in study region (Fig 3). A total of 24.7% of areas have historically experienced an increase in NDVI, but only 8.4% showed a progressively increasing trend, whereas 16.3% have been gradually stagnating in recent years. The areas with increasing and stagnation trend patterns were often intertwined and found in the centre of Qinghai and the west of Xinjiang Province. Significant decreasing trends occurred in 30.0% of the areas, with a particular concentration in the west of Qinghai Province as well as in some parts of northern Gansu Province and some northern and southern regions of Xinjiang Province.

### Historical climate trends over the study region

**Fig 4:**
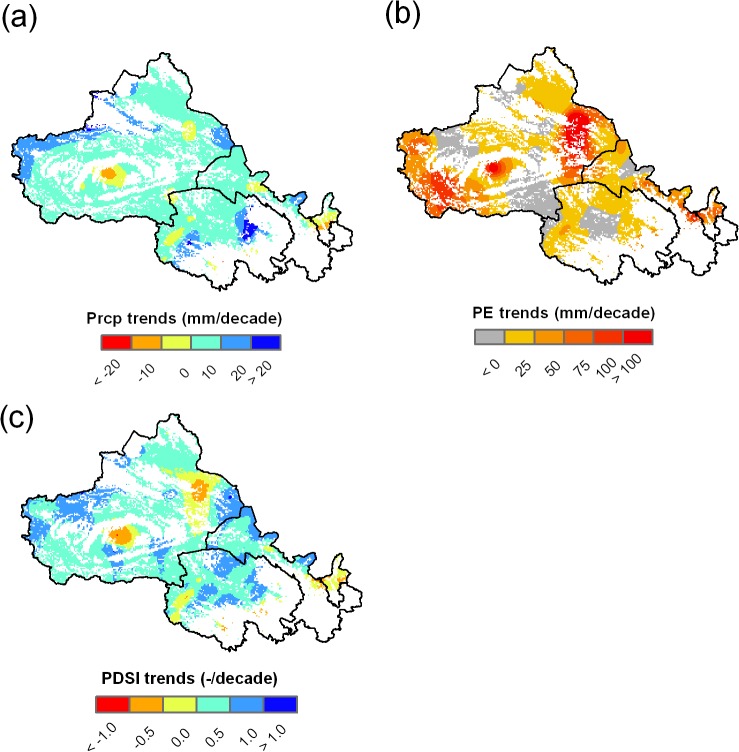
The linear time trends of Prcp, PE and PDSI over study period.

Fig 4 illustrates the linear time trend of Prcp, PE and PDSI for the study region. Progressively more Prcp was received in most areas of the study region, with a mean increase of approximately 10–20 mm per decade, except for the Ningxia and central region of Xinjiang where annual Prcp declined by 10–20 mm per decade (Fig 4A). PE showed a broad increase in the majority of the study region (Fig 4B). The PDSI incorporated Prcp and PE, showing a wetter climate in most of the study areas: the PDSI increased by 0.0–1.0 per decade except in Ningxia and some regions of Xinjiang Province where the PDSI decreased at a rate of 0.0–1.0 per decade (Fig 4C).

### NDVI relationships to PDSI and Year

**Fig 5:**
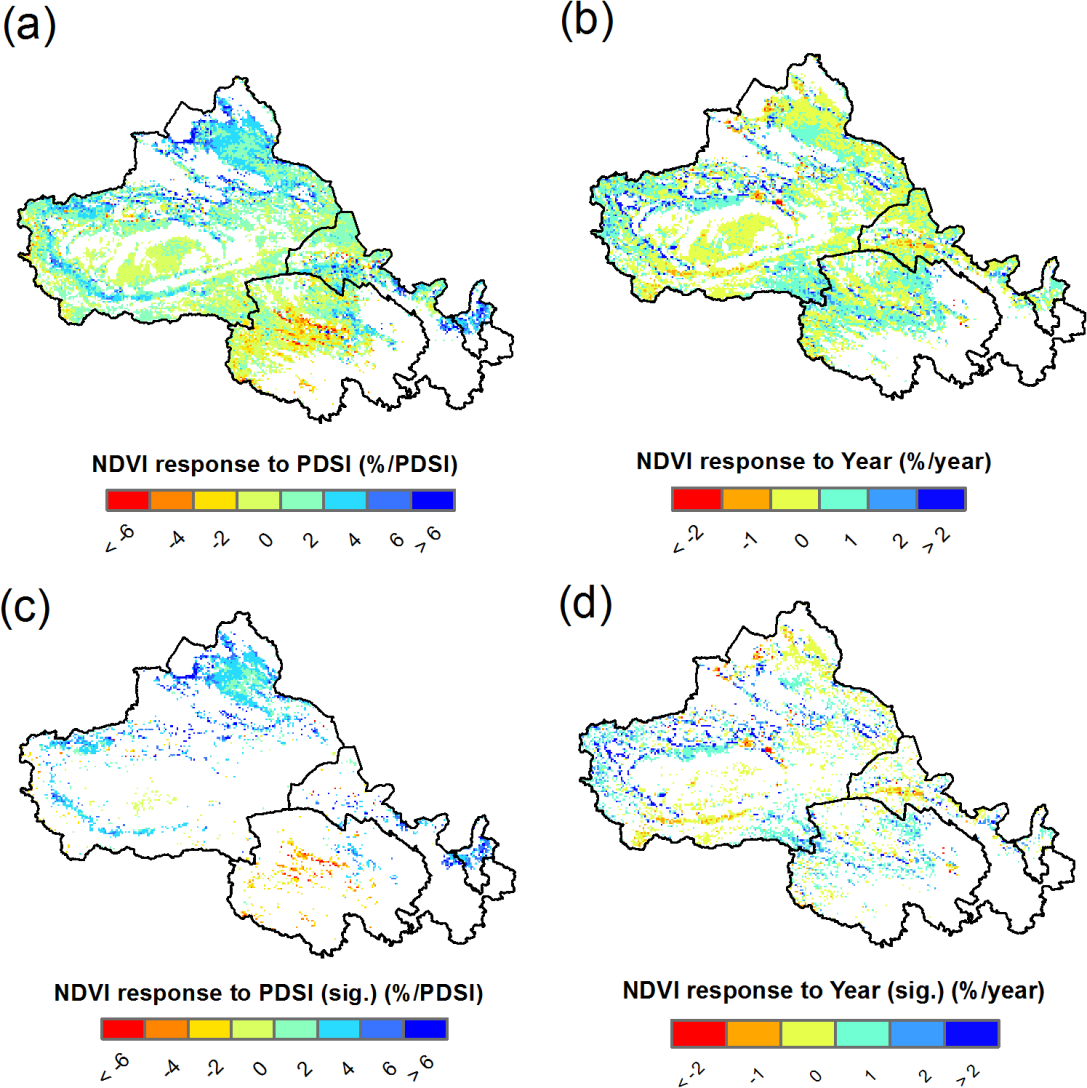
Response of the NDVI to PDSI and Year. All grids are plotted in (a) and (b), and only significant grids are plotted in (c) and (d). The base map is only for illustrative purposes.

**Fig 6:**
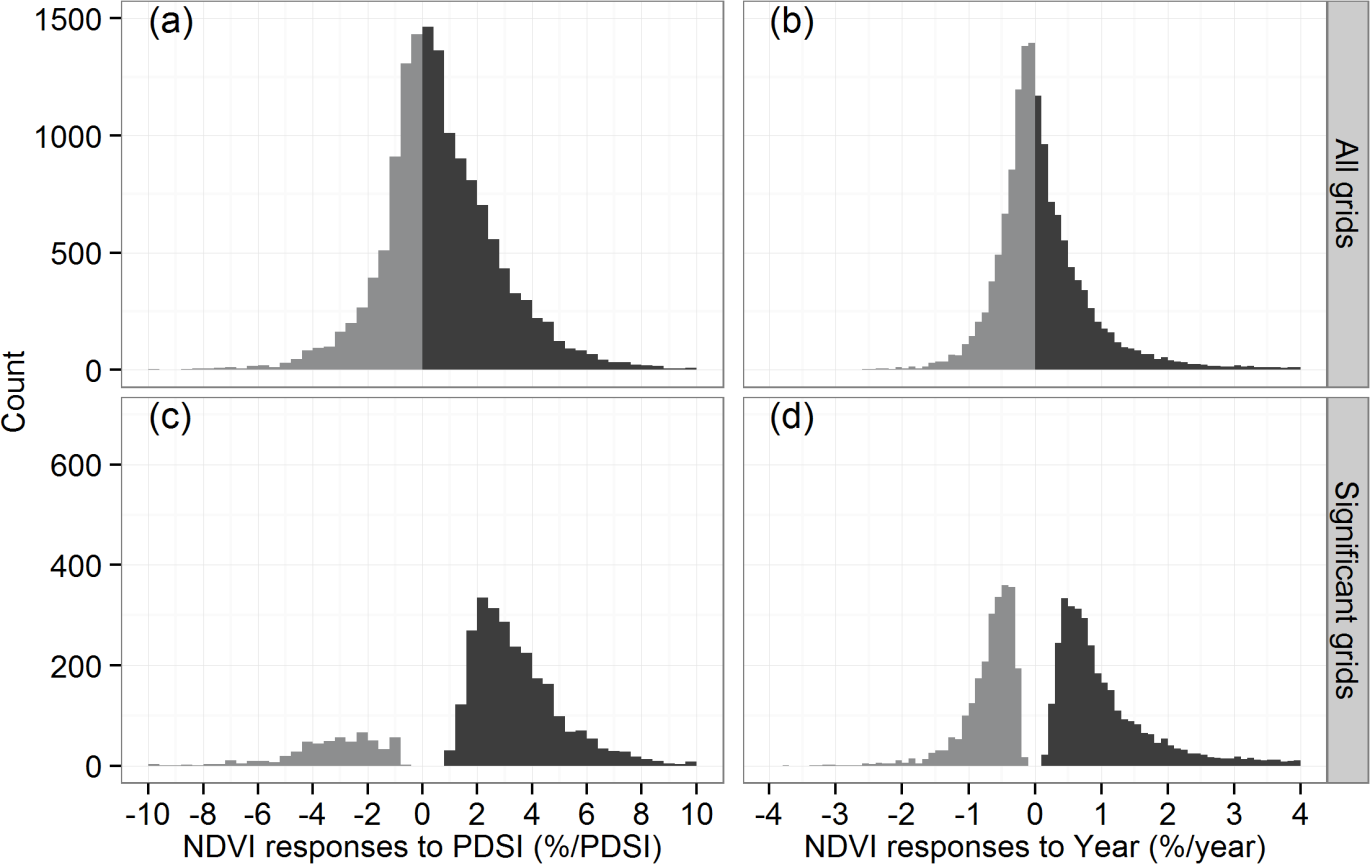
Histogram of NDVI responses to the PDSI and Year. All grids were plotted in (a) and (b), and only significant grids were plotted in (c) and (d).

The spatial distributions of the regression coefficients from the multiple regression model, i.e., the NDVI relationships to PDSI and Year, are illustrated in Fig 5, and the histogram is depicted in Fig 6, with a summary of statistics provided in Table 1. The results indicate that PDSI would positively associated with NDVI in the majority of the study area (Figs 5A and 6A), with significant positive relationships found in 18.1% of grids and significant negative relationships occurring in only 3.9% of grids (Table 1). Most positive relationships were found in the Xinjiang, Gansu and Ningxia Provinces with NDVI increasing 0–6% for each additional PDSI (Fig 5A). In contrast, significant negative relationships were found in the central Qinghai (Fig 5C). The regional average NDVI response to the PDSI is about 0.7% increase for each degree additional PDSI (Table 1).

**Table 1:** Summary of the NDVI response to the PDSI and Year and the changes in the NDVI due to the historical trends of the PDSI and Year from 1982–2008.

Variable	Neg_Sig^a^	Pos_Sig^a^	Average
NDVI responses to PDSI (%/PDSI)	3.9%	18.1%	0.7
NDVI responses to Year (%/Year)	16.9%	23.9%	0.2
NDVI changes by PDSI over 1982–2008 (%)	6.8%	15.2%	0.4
NDVI changes by Year over 1982–2008 (%)	16.9%	23.9%	4.0

^a^Neg_Sig/Pos_Sig: proportion of series showing significantly negative and positive regression coefficients (P<0.05).

Both positive and negative responses of the NDVI to Year were detected in the study region (Figs 5B and Fig 6B). The number of grids with a significant relationship was greater than those of the PDSI; approximately 23.9% of grids showed a significant positive relationship, and 16.9% showed a significant negative relationship. On regional average, the NDVI increased by 0.2% each year when the PDSI was held constant (Table 1).

### Estimating changes of the NDVI due to the historical PDSI and Year

**Fig 7:**
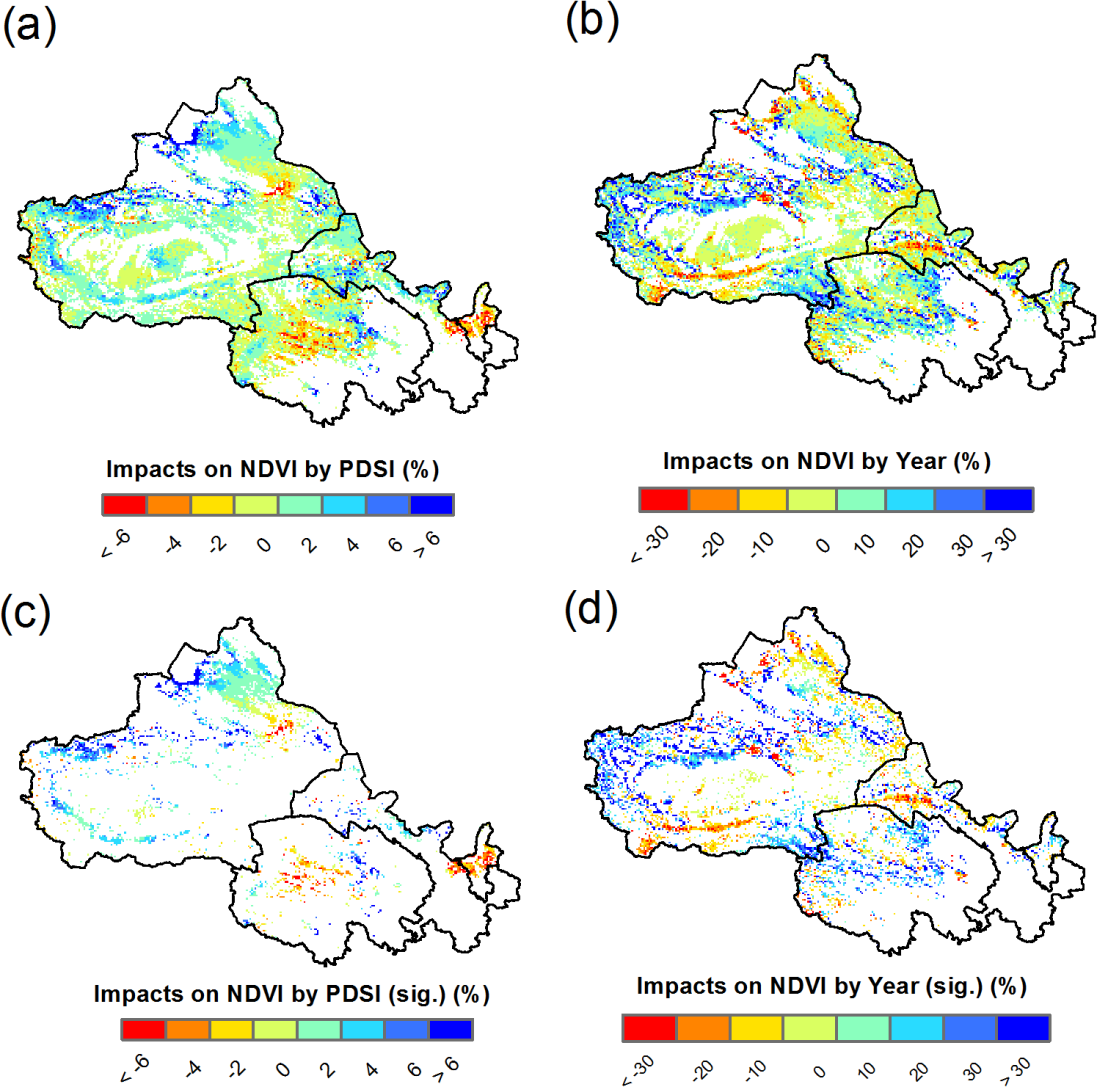
Estimated impacts on NDVI by historical trends in PDSI and Year. All grids were plotted in (a) and (b), and only significant grids were plotted in (c) and (d). The base map is only for illustrative purposes.

**Fig 8:**
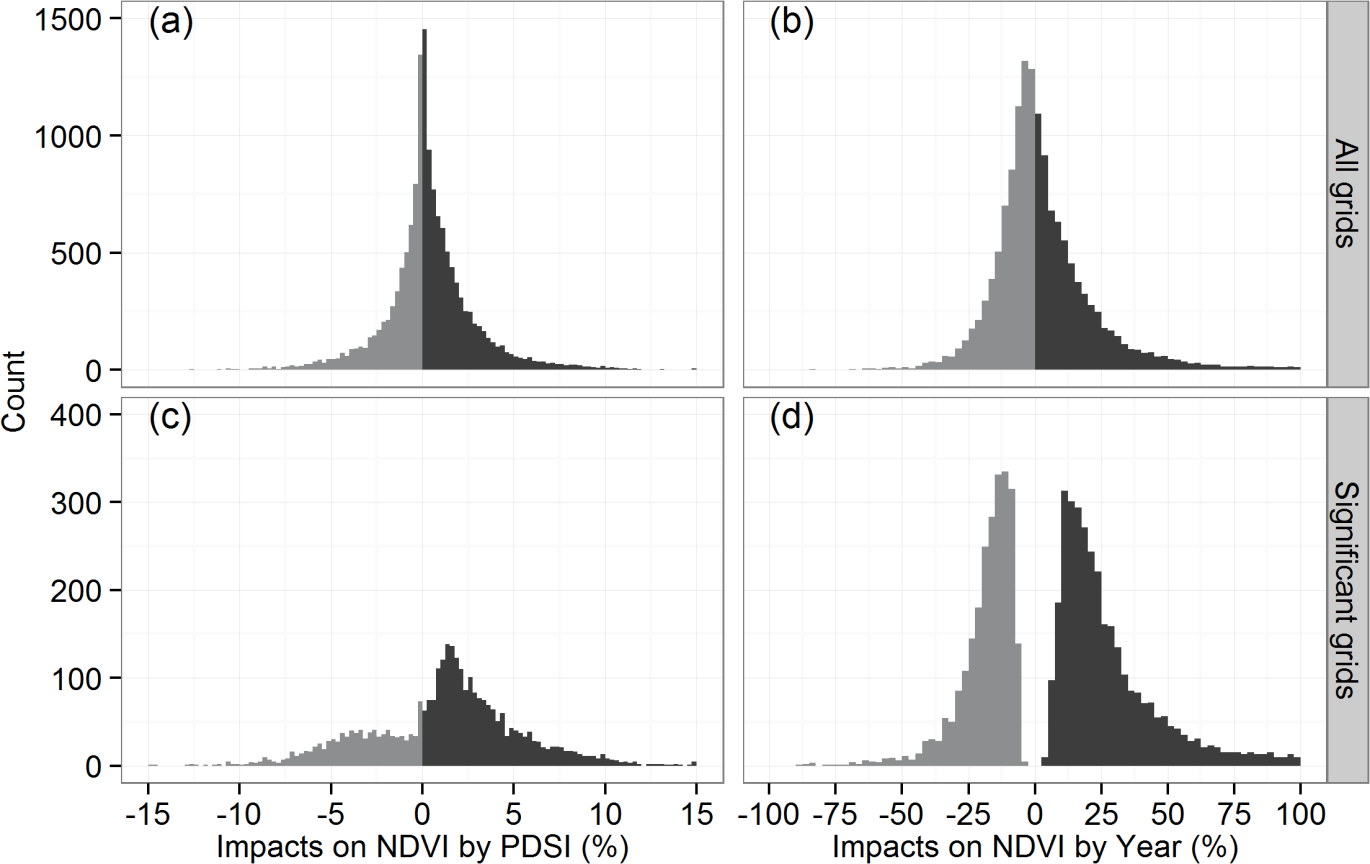
Histogram of estimated impacts on NDVI by historical trends in PDSI and Year. All grids were plotted in (a) and (b), and only significant grids were plotted in (c) and (d).

By inputting historical PDSI and Year in above multiple regression model, we estimated the individual impacts of PDSI and Year on NDVI over the period of 1982–2008. Our results suggest that the historical trends in the PDSI resulted in the improvement in the NDVI in most areas of the Xinjiang and Gansu Provinces (Fig 7A and 7C), with 15.2% showing a significant positive impact (Table 1). In contrast, some areas showed a declining NDVI as a result of the PDSI changes in Ningxia and central Qinghai Provinces (Fig 7A and 7C), with 6.8% exhibiting a significant negative impact (Table 1). The NDVI changes induced by the historical PDSI trends vary between approximately -15 and +15% (Fig 8A and 8C), and the average impact is approximately 0.4% from 1982–2008 (Table 1).

Holding the PDSI constant, the NDVI was estimated to increase by approximately 4.0%, on average, during the study period (Table 1). The increased NDVI due to the effect of Year was detected in the Qinghai and eastern region of Xinjiang Province (Fig 7B and 7D), while the NDVI declined over time in some areas of the south and north of Xinjiang Province (Fig 7B and 7D).

### Estimating changes in the NDVI grouped by each trend pattern

**Fig 9:**
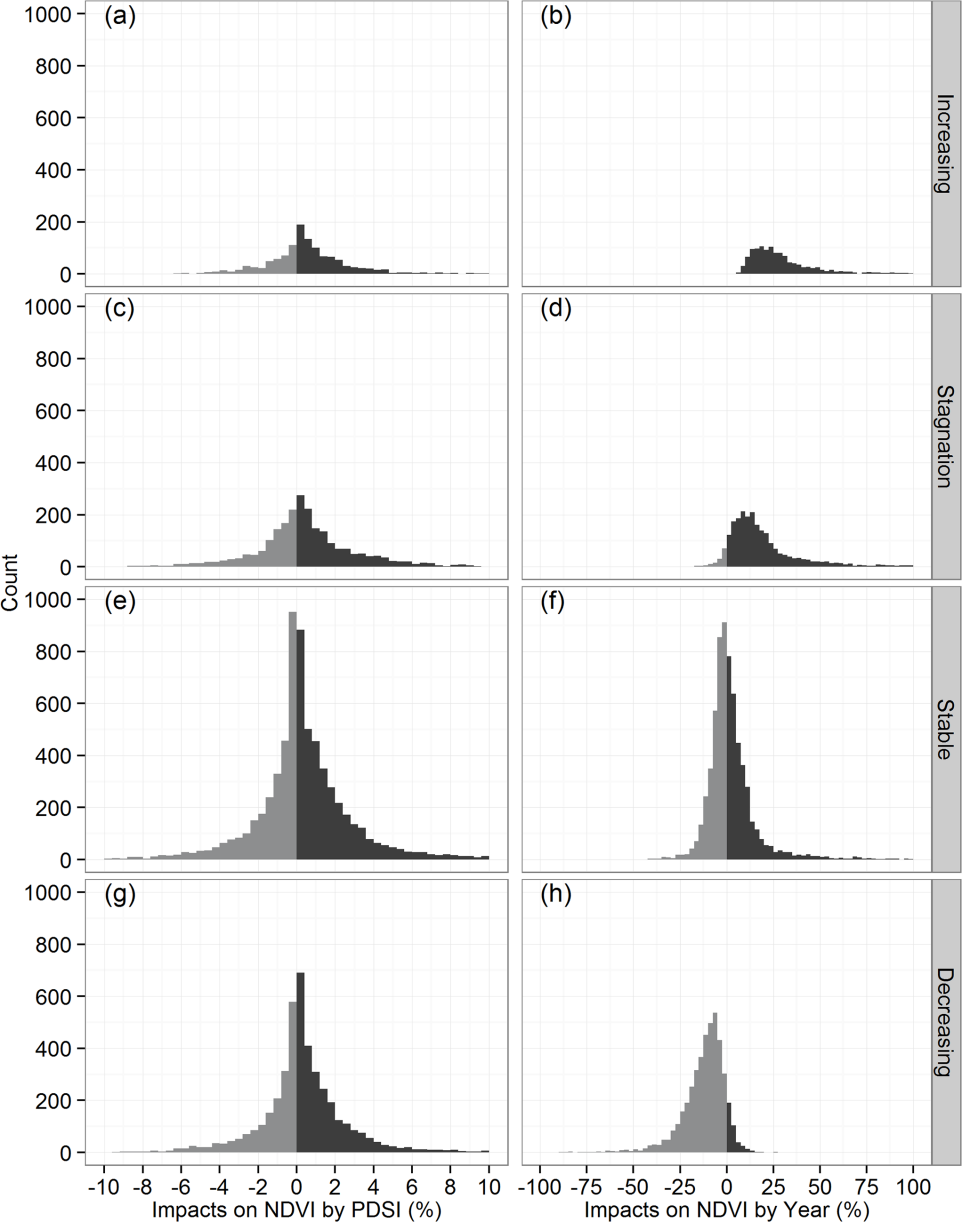
Impacts on NDVI by historical trends in PDSI and Year grouped by trend patterns.

Combining the above-described trend patterns and regression analysis, we compared the features of NDVI changes induced by the individual impacts of PDSI and Year in each trend pattern. The results suggest that the PDSI impacts on NDVI are very similar in each trend pattern (Fig 9 and Table 2). No significant difference was detected among trend patterns based on Tukey’s HSD analysis (Table 2).

**Table 2:** Summary of the changes in the NDVI for the individual trends of the PDSI and Year, grouped by each trend pattern, over the study period.

Trend pattern	PDSI			Year		
	Neg_Sig^a^ (%)	Pos_Sig^a^ (%)	Impact_avg_ ^b^ (%)	Neg_Sig^a^ (%)	Pos_Sig^a^ (%)	Impact_avg_ ^b^ (%)
Increasing	5.3%	11.9%	0.54a	0.0%	99.0%	33.5a
Stagnation	4.9%	14.2%	0.51a	0.0%	57.0%	22.2b
Stable	8.5%	17.5%	0.41a	7.4%	13.6%	2.7c
Decreasing	5.5%	13.2%	0.38a	45.4%	0.0%	-12.2d

^a^Neg_Sig/Pos_Sig: proportion of series showing significantly negative and positive regression coefficients (P<0.05).

^b^Impact_avg_: average impact on the NDVI by individual PDSIs and Years over the study region; values not sharing the same letter are significantly different (P<0.05).

In contrast, we detected a significant difference (P<0.05) on the NDVI changes by Year among the four trend patterns (Fig 9 and Table 2). With the PDSI held constant, the NDVI was increased by 33.5%, on average, during the study period for the increasing trend pattern, 22.2% for stagnation and 2.7% for stable and with declines of 12.2% for the decreasing pattern (Table 2).

## Discussion

Throughout the study region, approximately 45.3% of grids maintained a general stability over time, and 30.0% of grids showing a reduction in NDVI. In the remaining 24.7% of grids, the NDVI showed some increases, but the grids with a significant increase in vegetation only accounted for 8.4% of the total, whereas the NDVI in 16.3% of grids showed stagnation after an improvement in earlier years (Fig 3).

More specifically, from 1982–2008, a decreasing NDVI has been occurring in the west of Qinghai Province as well as in some parts of the north of Gansu Province and northern and southern regions of Xinjiang Province. Additionally, NDVI has been stagnating in recent years in the centre of Qinghai and the west of Xinjiang Province (Fig 3). Both of these findings do not support the results in earlier studies on expanding vegetative coverage [12] and declining desertification [5, 28] in China. Some reasons for this difference are the different underlying time frames considered among studies and the use of a linear time series model in these earlier studies. However, our findings agree with a recent study [11] that found NDVI tends to show an inverse trend after an earlier increase of vegetative coverage. Therefore, the identification of the major reasons for these decreasing and stagnating trends is crucial in creating an effective policy to combat the recent reduction of vegetative coverage.

Based on our multiple regression model, the response of the NDVI to the PDSI is positive in most grids, with 18.1% showing a significant positive relationship, which is more than the percentage of grids showing a significant negative relationship (only 3.9%) in our study region (Fig 5A and 5C). The positive relationship indicates that wetter (dryer) climate causes more (less) vegetative coverage, reflecting the influence of water conditions on the vegetation growth. However, it must be stressed that the historical changes of the PDSI only accounted for 0.4% of the improvement in the NDVI averaged over the study region (Table 1), with variability between -10% and +10% (Fig 7 and Fig 8). After controlling PDSI, we found that Year still played a major role in the variance of the NDVI compared with the PDSI. The NDVI was positively correlated with Year in 23.9% of grids in a significant manner, and the 16.9% of grids show a significant negative correlation (Table 1). On average, 4.0% of the improvement of the NDVI can be attributed to Year (Table 1), which is greater than the estimated NDVI impacts due to changes in the PDSI.

In addition, by contrasting their individual impacts in each trend patterns, we found that the historical trends of the PDSI is associated with a general increase in NDVI by 0.38–0.54% during the study period in the four trend patterns with no statistical significance difference among them (Fig 9 and Table 2). This finding reflects that the climate influences on each trend pattern are almost identical, thus not the major reason for the different trend patterns. In contrast, keeping the PDSI constant, a statistically significant difference was detected in the Year effect among the four trend patterns (Fig 9 and Table 2).

Therefore, our results do not support the major attribution of the NDVI changes to climate factors in previous studies [12, 29]. The reason for the disagreement is that the dominant climate impacts derived in the earlier studies were often obtained using a simple correlation analysis [29] or by graphically comparing the time trend of the NDVI and climate on the spatial scale [11], but the concurrence of an improved NDVI and a wetter climate does not necessarily indicate that all variance of the NDVI is caused by climate factors. In contrast, our results reflect the importance of non-climatic impacts on the NDVI trends, as quantified by the proxy indicator of Year in the multiple regression model.

Recent studies focusing on specific deserts in China have identified the importance of some non-climatic factors [30 – 32]. For example, using Landsat-5 data, Ge et al. [31] compared the importance of Prcp and land use in the Naiman Banner Desert, finding that the impact of land uses is often 8–20 times more dominant than that of Prcp on land degradation. Based on an experiment carried out in Maduo County in Qinghai, Zhang et al. [33] found that overgrazing presents a strong influence, causing considerable losses of soil organic matter and total nitrogen, two indicators of environmental degradation. By contrasting a numeric model simulation and MODIS satellite data in the Heihe River Basin in Gansu, Zhou et al. [32] found that 90.5% of the contributions to desertification were related with human activities rather than climate factors.

In view of these results, we conclude that the major driver for vegetative coverage in Northwest China is not associated with climate, but we have insufficient information available to assess the actual major non-climatic drivers. To conduct such an analysis, it is necessary to have high-resolution data on all key potential drivers of non-climatic factors, especially for human activities. To our knowledge, such a dataset does not exist on the grid scale in our study, which prevents us from including more specific non-climatic factors in statistical model. A further investigation is needed to address the major drivers for changes in vegetative coverage by the use of a newly developed county-level agricultural socioeconomic database in China [34].

## Conclusions

By combining trend patterns and a multiple regression analysis, this study examined the impacts of climate and non-climatic factors on NDVI trends in the desert areas of Northwest China. Examining the period from 1982–2008, our results show that approximately 24.7% of the study region exhibited an overall improved NDVI; 16.3% among the region has showed stagnation in recent years and only 8.4% had a significantly increasing trend. In addition, 45.3% of the area has maintained stability and 30.0% has been declining over time. This result differs from the current viewpoint of NDVI reported by previous studies. Our trend pattern analysis, combined with the results of the multiple regression model, showed that there was no significant difference in the PDSI impacts on NDVI among the four trend patterns. This finding indicates that climatic impacts on each trend pattern are similar and thus not considered the major reason for the different trend patterns. However, after controlling PDSI, a statistically significant difference was detected for the Year impact among the trend patterns. Therefore, we mainly attribute the different trend patterns of the NDVI to non-climatic factors rather than climate. An identification and comparison of the importance of specific non-climatic factors based on high-resolution data in a future investigation would be beneficial in creating an effective plan to combat the recent desertification trend in China.

## Supporting Information

S1 Dataset NDVI and climate data. (ZIP)

S1 Fig Examples of trend patterns of the NDVI. (TIF)

S1 File Trend pattern analysis of the NDVI. (PDF)
