# Supplementary figures and images for: Trend Patterns of Vegetative Coverage and Their Underlying Causes in the Deserts of Northwest China over 1982 – 2008
Source: PLoS One. 2015 May 11;10(5):e0126044. doi: 10.1371/journal.pone.0126044 (PMC4427295; doi:10.1371/journal.pone.0126044)

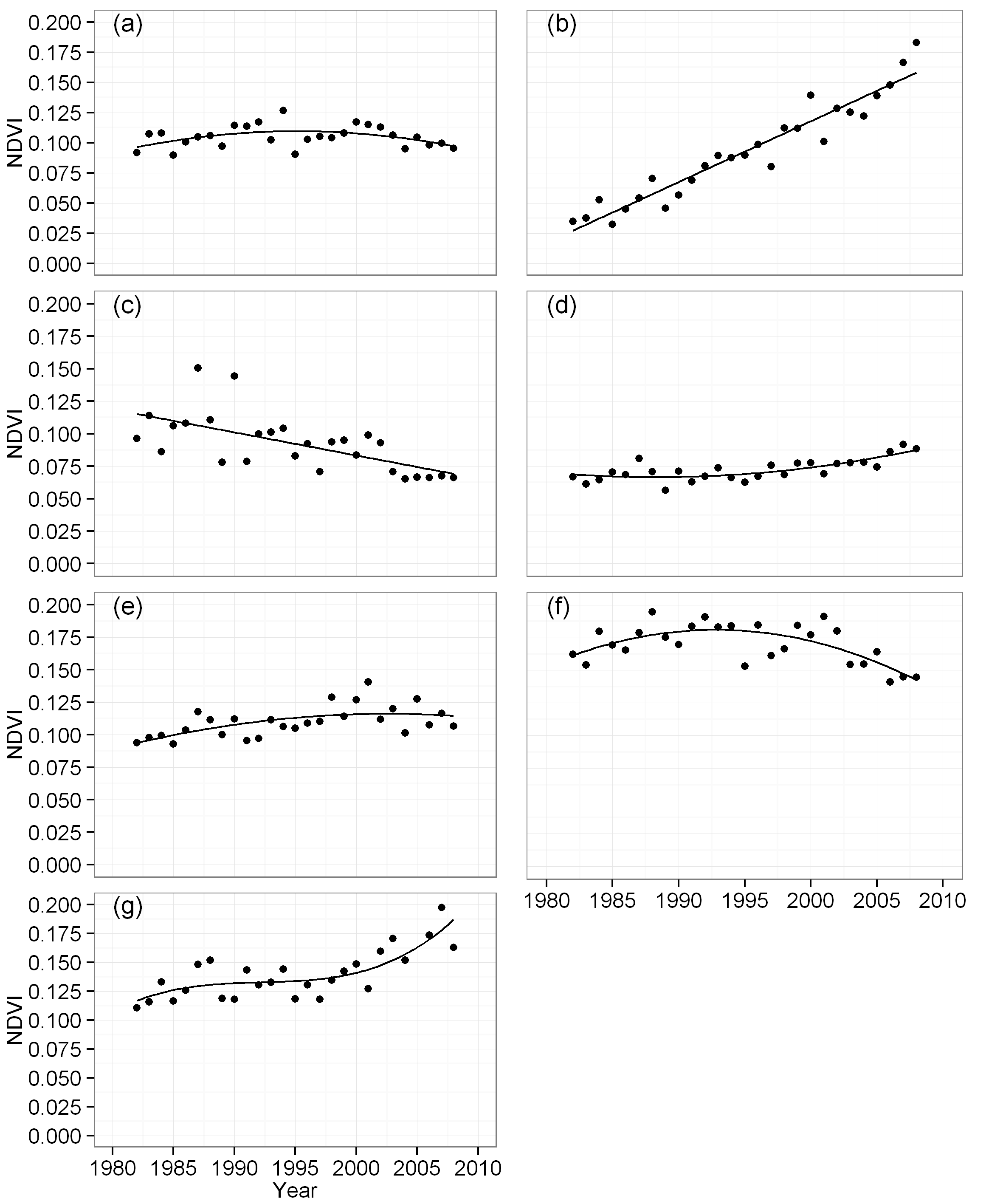

Supplement: S1 Fig — (TIF) [file pone.0126044.s002.tif]
