# Supplementary material for: Trend Patterns of Vegetative Coverage and Their Underlying Causes in the Deserts of Northwest China over 1982 – 2008
Source: PLoS One. 2015 May 11;10(5):e0126044. doi: 10.1371/journal.pone.0126044 (PMC4427295; doi:10.1371/journal.pone.0126044)
Supplement: S1 File — (PDF) [file pone.0126044.s003.pdf]

S1 File. Trend pattern analysis of the NDVI

We classified the NDVI trends into four trend patterns: increasing, stagnation, stable and decreasing. An increasing pattern denotes a significantly increased trend of the NDVI over time; a stagnation pattern denotes that the NDVI has been stagnating or declining in recent years despite an overall improvement; a stable pattern denotes no clear change over time; and a decreasing pattern denotes a declining NDVI.

The classification algorithm we used is similar to that of Ray et al. [1]. To classify trends, four polynomial functions of the NDVI-time relationship were fitted for each grid, i.e., an intercept-only model (Equation 1), a linear model (Equation 2), a quadratic model (Equation 3) and a cubic model (Equation 4). These models are as follows:

$$\begin{cases} NDVI = k & (1) \\ NDVI = at + k & (2) \\ NDVI = at^2 + bt + k & (3) \\ NDVI = at^3 + bt^2 + ct + k & (4) \end{cases}$$

where t denotes year; a, b and c are the regression coefficients; and k is the intercept.

Then, using the Akaike Information Criterion (AIC; Equation 5), proposed by Akaike [2], we calculated the best-fitted model for the NDVI trend:

$$AIC = n\log(\frac{SS}{n}) + 2\rho, \qquad (5)$$

where SS is the residual sum of squares, n is the sample size and p is the number of parameters. The model with the lowest AIC was considered as the best

representation of the NDVI trend for the given grid.

Using the lowest AIC model, we tested the model's goodness of fit based on the F statistic against the null hypothesis of the constant model ($P<0.05$).

When the AIC criterion suggested an intercept-only model, this implied that the NDVI had a "stable" trend pattern. In addition, if the F-test failed for any model, this meant that no model could represent the observations, and we reassigned these grids into the "stable" category. An example of first determining a quadratic model but finally reclassifying it as a "stable" trend is demonstrated in Fig. S1a.

S1 Fig. Examples of trend patterns of the NDVI.

When the AIC criterion indicated a linear model, we classified the NDVI trend based on the sign of the slope. If the slope was positive, it meant that the NDVI was increasing, and thus we classified it into the "increasing" category (Fig. S1b). If the slope was negative, it meant that the trend was decreasing; we classified this as a "decreasing" pattern (Fig. S1c).

When the best-fit model was quadratic, we first determined the sign of the quadratic term. If the quadratic term was positive, it meant that the NDVI was "increasing" (Fig. S1d). When the quadratic term was negative, it meant that the NDVI had a peak and was thus classified as "stagnation" (Fig. S1e). However, when the projected year of the peak was beyond the year 2010 (i.e., two more years from the end year of the NDVI observation), it only meant that the NDVI had not reached its peak; thus it was reclassified as "increasing." However, if after a peak, the NDVI reached a very low level, it meant that the NDVI had decreased (Fig. S1f). We

considered these cases as “decreasing” and tested them by determining whether the average NDVI in the period of 2004–2008 was lower than the average of the years 1982–1986.

When the cubic model was chosen, we calculated both the year when the NDVI was the lowest and the year of the peak. If the projected year of the peak was reached prior to 2010, we classified it as “stagnation,” and after 2010, it was classified as “increasing” (Fig. S1g). In some cases, the NDVI declined after a peak, followed by a minimum in recent times and then another increase. We reclassified such cases as “increasing.” Additionally, if the NDVI for 2004–2008 had reached the level of 1982–1986, we reclassified the trend into the “decreasing” category.

References

1. Ray DK, Ramankutty N, Mueller ND, West PC, Foley A. Recent patterns of crop yield growth and stagnation. Nat Commun 2012; 3: 1293.
2. Akaike H.. A new look at the statistical model identification. IEEE Transactions on Automatic Control 1974; 19: 716–723.
