# Supplementary material for: Trend Patterns of Vegetative Coverage and Their Underlying Causes in the Deserts of Northwest China over 1982 – 2008
Source: PLoS One. 2015 May 11;10(5):e0126044. doi: 10.1371/journal.pone.0126044 (PMC4427295; doi:10.1371/journal.pone.0126044)
Supplement: S1 Dataset — (ZIP) [file pone.0126044.s001.zip › S1_data/Readme.doc]

**S1 data. NDVI and climate data**

The annual average NDVI dataset were available here.

demNW.txt: the base map we are using. It records the elevation of our study region.

NDVI folder: it is the raw data including three columns; Row&Col, Year, NDVI.

Row&Col: The location marker indicates the number of row (the first three digits) and column (the last three digits) in demNW.txt map.

Year: the Year data

NDVI: the annual average NDVI data.

For the climate data, they were provided by the China Meteorological Administration and we don’t have right to distribute the data based on the agreement we signed with them. But data is available after registration on their website (cdc.cma.gov.cn). Or we can also contact China Meteorological Administration for the readers interested in. In the case, please contact Dr. Tianyi Zhang (zhangty@mail.iap.ac.cn).
